# Effect of Bacteria on the Wound Healing Behavior of Oral Epithelial Cells

**DOI:** 10.1371/journal.pone.0089475

**Published:** 2014-02-21

**Authors:** Rupa Bhattacharya, Fanxing Xu, Guangyu Dong, Shuai Li, Chen Tian, Bhaskar Ponugoti, Dana T. Graves

**Affiliations:** 1 Department of Periodontics, School of Dental Medicine, University of Pennsylvania, Philadelphia, Pennsylvania, United States of America; 2 School of Life Science and Biotechnology, Dalian University of Technology, Dalian, China; 3 Department of Implant Dentistry, Peking University, School and Hospital of Stomatology, Beijing, China; University Hospital Hamburg-Eppendorf, Germany

## Abstract

Wounded tissue offers opportunity to microflora to adhere, colonize, invade and infect surrounding healthy tissue. The bacteria of the oral cavity have the potential to alter the wound healing process by interacting with keratinocytes. The aim of this study was to investigate mechanisms through which oral bacteria may influence re-epithelialization by interacting with gingival keratinocytes. By an *in vitro* scratch assay we demonstrate that primary gingival keratinocytes have impaired closure when exposed to two well characterized oral bacteria, *P. gingivalis,* and to a lesser extent, *F. nucleatum*. *P. gingivalis* reduced wound closure by ∼40%, which was partially dependent on proteolytic activity, and bacteria was still present within infected cells 9 days later despite exposure to bacteria for only 24 h. Both oral bacteria caused keratinocyte apoptosis at the wound site with cell death being greatest at the wound edge. *P. gingivalis* and *F. nucleatum* adversely affected cell proliferation and the effect also had a spatial component being most striking at the edge. The impact of the bacteria was long lasting even when exposure was brief. Cell migration was compromised in bacteria challenged keratinocytes with *P. gingivalis* having more severe effect (p<0.05) than *F. nucleatum*. Quantitative real time PCR of bacteria challenged cells showed that *P. gingivalis* and to a lesser extent *F. nucleatum* significantly downregulated cell cycle genes cyclin1, CDK1, and CDK4 (p<0.05) that are critical for GI/S transition. Further, genes associated with cell migration such as integrin beta-3 and -6 were significantly downregulated by *P. gingivalis* (p<0.05).

## Introduction

The gingiva is lined by stratified squamous epithelium that is an interface between the external environment and underlying connective tissue. Wounding of the gingiva involves disruption of this barrier function. Healing involves proliferation, migration, and differentiation of epithelial keratinocytes situated at the edge of the wound [1]. Wounds can be broadly categorized as having either an acute or a chronic etiology. Chronic wounds have delayed healing and frequently have an endogenous factor that compromises the healing process [1]. Chronic wounds are often colonized by bacteria [2]. Bacteria play an active role in wounds [3]. Further, anaerobes are found to form a significant proportion of the microbial population in chronic wounds [4].

The normal microflora of the oral cavity is diverse and abundant. The oral cavity is initially cultivated by Gram positive bacteria, and later shifts Gram-negative anaerobes, particularly in subgingival plaque [5]. The gingival epithelium lies at the interface between the external environment and functions as the primary physical barrier to infections by oral bacteria. Two anaerobic oral bacteria that have been the subject of extensive study are *Porphyromonas gingivalis* and *Fusobacterium nucleatum* and are found in close contact with the epithelium of the gingiva [5], [6]. Recently entry of *P. gingivalis* into systemic tissue via periodontal tissues has been cited [7]. The interaction between these bacteria and host cells including gingival keratinocytes has been well studied in relationship to the host response and inflammation but not in processes such as wound healing [5], [8], [9].

An essential feature of oral healing is restoration of an intact epidermal barrier through re-epithelialization. The directed migration of keratinocytes as well as proliferation and survival are critical to wound re-epithelialization. Information regarding the impact of oral bacteria on re-epithelialization by oral keratinocytes is lacking even though it is likely to be important in response to trauma as well as the healing process that follows viral infection that causes ulcerative oral lesions and in response to oral and periodontal surgery [10]–[12]. *P. gingivalis* and *F. nucleatum* are commonly found oral pathogens in humans. The aim of this study was to investigate the impact of two different oral bacteria on re-epithelialization under well controlled conditions and to investigate potential mechanisms that may be affected, keratinocyte migration, proliferation and apoptosis. The results indicate that both bacteria impede the normal re-epithelialization process even when transiently exposed to keratinocytes, that there is a spatial aspect to this impact and that it occurs through mechanisms that primarily involve migration but also involve reduced proliferation along with enhanced apoptosis.

## Materials and Methods

### Cells and Bacteria

Primary gingival keratinocytes were kindly provided by Dr. Kinane, School of Dental Medicine, University of Pennsylvania, PA from gingival tissue biopsies that were obtained with written informed consent from periodontally healthy patients undergoing oral surgical procedure at the University of Pennsylvania’s School of Dental Medicine with Institutional Review Board approval. Primary gingival keratinocytes were cultured in Derma K Life medium (Lifeline cell technology, Walkersville, MD) with all additives except insulin. Anaerobic bacteria *Porphyromonas gingivalis* strain (ATCC 33277) and *Fuscobacterium nucleatum* (ATCC 25586) were purchased from ATCC. Both strains of bacteria were grown in GAM medium (Nissui pharmaceuticals, Tokyo, Japan) under oxygen deprived anaerobic conditions as described by Brozovic *et al*., [13].

### *In vitro* Wound Assay

Primary gingival keratinocytes were cultured in collagen coated eight chambered slides (Nunc, Rochester, NY) and maintained in KBM-2 medium. For creating wound, confluent monolayer of keratinocytes were scratched with a P200 micropipette tip (GeneMate, ISC BioExpress, Kaysville, UT) and challenged with bacteria at MOI of 10∶1 (bacteria : keratinocytes) under standard culture conditions. Bacteria were removed after 24 h and thereafter cells were maintained in medium containing antibiotics of penicillin and streptomycin (Life Technologies, Grand Island, NY). In some experiments bacteria and keratinocytes were incubated with 100 nM leupeptin (Sigma-Aldrich, St Louis, MO) for 24 h. Images of the scratch assay were captured with an inverted microscope at three regions per scratch. Images were taken immediately after scratch and then on later time points per the requirements of the experiment. Gap distance between the two margins of wound was measured with Image Pro Plus 7.0 software (Mediacybernetics, Silver Spring, MD). No gap remaining between the two edges of the wound was considered as complete closure.

For apoptosis and cell proliferation studies, primary oral keratinocytes were seeded in four chambered slides (Labtek, Bioexpress, Kaysville, UT) and maintained in Derma K Life medium. Scratch followed by bacteria challenge was carried out as mentioned above. Cells were incubated with bacteria for 24 and 48 h followed by fixing cells for immunocytochemistry assays of TUNEL and PCNA staining.

### *P. gingivalis* Internalization, TUNEL and Immunocytochemistry Assays

To examine bacteria internalization within keratinocytes, oral bacteria were labeled with carboxyfluorescein diacetate N-hydroxysuccinimidyl ester (CFSE, Ebioscience, San Diego, CA). Keratinocytes were incubated with labeled bacteria (MOI 1∶10) in 8 chambered slides at 37°C for 24 h with or without 100 nM leupeptin. After 24 h cells were rinsed thoroughly and incubated with antibiotics for the indicated amount of time. After fixation cells were permeablized with 0.1% triton X-100 and F-actin was stained with phalloidin-Texas red (Invitrogen) for 20 minutes and then mounted with DAPI. Fluorescent images were captured using Nikon Eclipse 90 i microscope. For internal localization of *P. gingivalis* or *F. nucleatum* fluorescent deconvolution microscopy was performed with images taken along the Z axis in 2 µm increments over 10 µm. Each keratinocyte as examined in the Z axis in 2 micron sections and internalization was determined by co-localization with F actin since F actin is only found in the cytoplasm. The cytoplasm adjacent to the cell membrane was excluded from analysis and only bacteria that co-localized with F-actin were counted. Total of 30–50 cells per 5 fields per well with 3 wells per experiment were examined. Experiments were performed at two to three times with similar results. Apoptosis of cells following the *in vitro* scratch assay was assessed by fluorescence microscopy using DeadEnd Fluorometric TUNEL system (Promega, Madison, WI) following the manufacturer’s instructions. To assess proliferation keratinocytes were examined for proliferating cell nuclear antigen (PCNA) by immunocytochemistry with PCNA antibody (Santa Cruz, Dallas, TX) (200 µg/ml) and localized with biotinylated secondary antibody (Chemicon, Billerica, MA ) followed by incubation with streptavidin Alexa 546 (Invitrogen, Grand Island, NY ). Cells were washed with PBS and mounted with DAPI (Invitrogen). For TUNEL and PCNA cells were examined in at least five different fields per slide using NIS-elements AR software in a blind fashion. Briefly, for cell proliferation cells that showed co-localization of PCNA staining with DAPI were counted as immunopositive. For apoptosis, cells staining for TUNEL were counted as positive. The total number of cells in the scratched region was counted based upon DAPI staining of nuclei. Cell proliferation or apoptosis was then expressed as ratio of number of positive cells per total number of cells for a particular region.

Scratch assay cells incubated with bacteria were assayed for apoptosis using Annexin V antibody. Briefly, cells were incubated with biotinylated Annexin V antibody (Life technologies, Grand Island, NY) for 5 minutes and were then washed with binding buffer (0.1 M Hepes pH 7.4, 1.4 M NaCl and 25 mM CaCl_2_) before fixing with 2% formaldehyde. Cells were then incubated for 15 minutes with avidin-fluorescein (Life technologies, Grand Island, NY), followed by PBS washes and mounting with DAPI. Images were captured with Nikon Eclipse 90 i microscope using filter set for FITC. Cells with green staining of cell membranes were considered positive for apoptosis.

For assessing expression of integrin beta-3 protein, keratinocytes were incubated in the chamber slides with control or P. *gingivalis* or F. *nucleatum* at MOI 1∶10 for 72 h and then followed by immunofluorescence staining with an antibody to integrin beta-3 (Santa Cruz, Dallas, TX), secondary antibody (Vector Laboratories, Burlingame, CA) and DAPI. Immunofluorescence was captured using Nikon fluorescence microscopy ECLIPS 90 i and analyzed with by Nikon fluorescent microcopy using Nikon NIS-Elements software. Quantitation of integrin beta-3 at the protein level was determined by measuring fluorescence intensity of integrin beta-3 normalized to DAPI. The average of fluorescence intensity measured for each individual cells divided by total number of cells in a field was used to find the mean fluorescence intensity (MFI).

### Transwell Migration Assay

Primary oral keratinocytes were incubated with bacteria overnight at a MOI of 1∶10. Keratinocytes (2×10^5^) were transferred to the upper chamber of a Transwell migration chamber (Costar 3422, Corning, Tewksbury, MA) with a polycarbonate membrane (pore size 8.0 µm) at 37°C. After 6 h of incubation, cells were removed from the top of the membrane by gently swabbing and the membrane was mounted with DAPI mounting medium. Migrated cells on the other side were counted by Nikon NIS elements software under fluorescence microscope.

### mRNA Analysis

Primary human gingival epithelial cells were cultured in 6-well plates (Corning Lifesciences, Tewksbury, MA). Cells were then incubated with *P. gingivalis* or *F. nucleatum* at MOI 1∶10 for 24 and 72 h. RNA was extracted from cells using RNeasy mini kit (QIAGEN GmbH, Germany). Total RNA was used for cDNA synthesis using a kit from Life Technologies (Carlsbad, CA). Real-time PCR was carried out using fluorescent probes from (Roche Applied Science, Indianapolis, IN). The expression of each gene was quantitated on day 0, 1 and 3. Housekeeping gene ribosomal protein L32 was used to normalize the expression of each gene for each of the time point. The values of δδCt from three separate experiments were combined to obtain the mean and standard error of the mean. For the convenience of representation the expression of a gene on day 0 is taken as 1, and the expression on day 1 and 3 is represented relative to expression on day 0.

Gene expression of TNFα and IL6 was studied by incubating cells with *P. gingivalis* or *F. nucleatum* at MOI 1∶10 for 24 h, followed by RNA extraction and RT PCR. Expression of gene was quantitated against untreated control.

### Statistical Analysis

In all assays, data collected from three independent experiments were subjected to statistical analysis by one way ANOVA using Tukey’s test for comparison between groups with level of significance set at p<0.05.

## Results

### Oral Pathogens cause Delayed Wound Healing in Oral Keratinocytes

**Figure 1:**
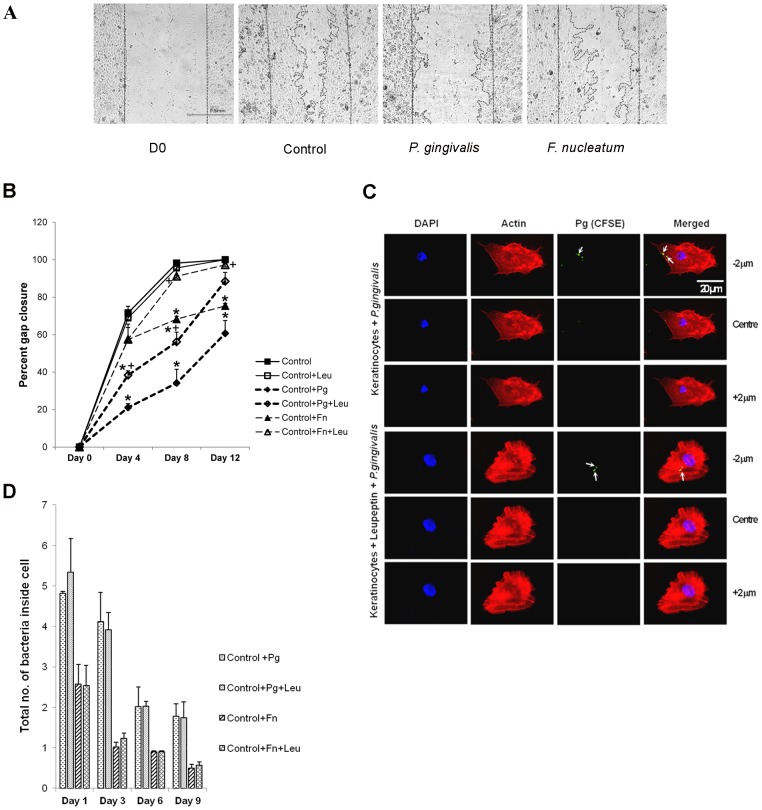
Delay in gap closure in pathogen infected scratch. **A.** Scratch assay for assessing wound healing in periopathogen challenged primary gingival keratinocytes. The scratch width at the beginning of the scratch assay is represented by D0, wound healing on day 4 is represented in cells with no treatment (control), *P. gingivalis* and *F. nucleatum* challenge. Straight line represents the initial scratch boundary while curved line represents the leading edges of the healing wound. **B.** Graph shows scratch assay in oral primary keratinocytes where gap filling was measured based on the distance between the leading edges of the scratch. The data is average ± SEM from three independent experiments and shows ANOVA using Tukey HSD test. Significant difference between control and bacteria treatment is represented by *p<0.05. Significant difference between bacteria alone and bacteria along with Leupeptin treatment for a time point is represented by +p<0.05. **C.** CFSE based fluorescence of *P. gingivalis* co-localizing with actin was used to confirm presence of bacteria inside keratinocytes incubated without and with leupeptin. Bacteria inside keratinocyte is indicated by arrows. **D.** Graph represents the number of bacteria found within infected keratinocytes over period of 9 days thereby indicating the internalization of bacteria in medium without and with leupeptin. Values represent the average from three independent experiments.

Two different oral bacteria were examined in an *in vitro* “scratch” re-epithelizalization assay to assess the direct effect on primary cultures of oral keratinocytes. Incubation of cells with *P*. *gingivalis* delayed closure of the gap as evidenced by fewer cells that migrated into the denuded area while *F. nucleatum* had less of an effect (Figure 1A). For quantitative analysis the percent closure of the gap was measured. On day 4 *P*. *gingivalis* reduced the extent of closure by 70% compared to untreated cells (p<0.05) (Figure 1B). On day 8 the gap was largely closed in the control group and the degree of closure was reduced by 65% in cells incubated with *P. gingivalis* (p<0.05) (Figure 1B). *F. nucleatum* reduced closure by 20% on day 4, 30% on day 8 (p<0.05) and by 24% on day 12 (p<0.05) compared to untreated cells (Figure 1B). The greater effect of *P*. *gingivalis* on delayed closure compared to the effect of *F. nucleatum* was significant on days 4 and 8 (p<0.05). It should be noted that the long term effect caused by exposure to bacteria at later time points occurred despite the fact that bacteria were rinsed away after incubation with keratinocytes for 24 h and cells were subsequently incubated in media containing antibiotics.

To assess whether re-epithelialization of the denuded surface was due to bacterial proteases [14], [15] cells were incubated in medium containing a known inhibitor of serine and cysteine proteases, leupeptin and concomitantly challenged with bacteria. Leupeptin alone had no effect in the absence of bacteria demonstrating that endogenous serine/cysteine proteases were not vital to gap closure. However, leupeptin significantly reduced the negative effect of *P*. *gingivalis* on gap closure by 45% and 39% (p<0.05) on days 4 and 8 respectively (Figure 1B). In comparison leupeptin had no effect on *F. nucleatum* modulated closure. Because the effect of bacteria exposure was considerable and long-lasting we determined whether oral bacteria were maintained intracellularly over an extended period of time. CFSE based staining of pathogen along with actin co-localization studies revealed the intracellular existence of pathogen (Figure 1C). Intracellular *P*. *gingivalis* and *F. nucleatum* were detected over a time course of 9 days (Figure 1D). We also determined that leupeptin did not affect *P*. *gingivalis* uptake (Figure 1D). Thus, the prolonged effect of oral bacteria may be related to its intracellular localization.

### Bacteria Challenged Cells Exhibit Apoptosis

**Figure 2:**
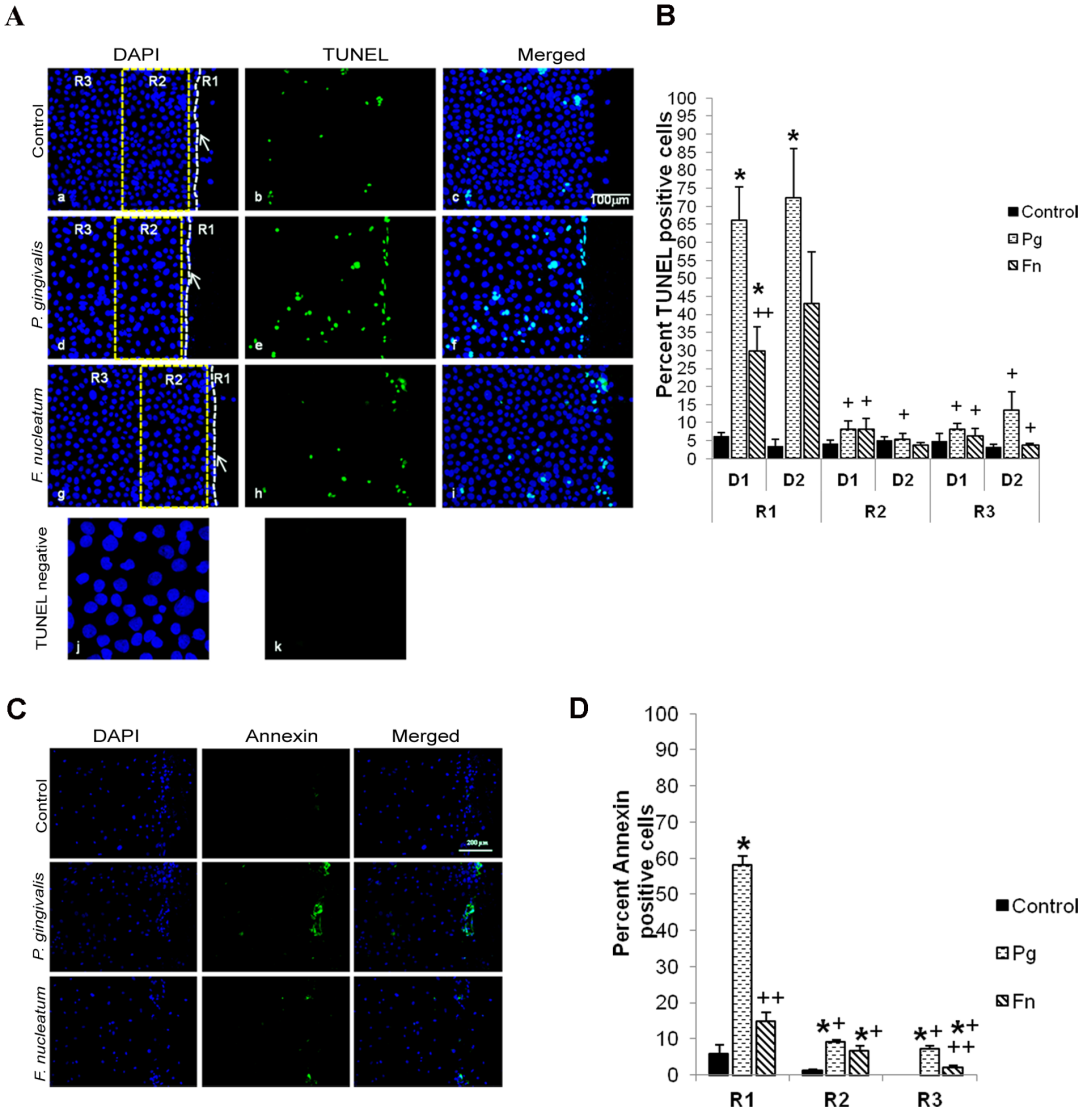
Assessment of apoptosis in cells undergoing scratch assay. **A.** Primary oral keratinocytes subjected to scratch assay and challenged with bacteria were stained for apoptosis using TUNEL kit (b, e and h). Nuclei was stained with DAPI (a, d and g). Arrow indicates the edge of the scratch. The regions of the scratch as used for spatial analysis in apoptosis is marked with lines, R1 = scratch edge, R2 = area adjacent to scratch edge and R3 = at least 200 µm away from the scratch edge. For representation purpose region R3 is not to scale. The negative control on bacteria challenged scratch assay for TUNEL was done by staining without rTdT enzyme and is represented by (k). **B.** Graphical representation of the number of TUNEL positive cells divided by total number of cells and expressed in percent at different regions of the scratch and at different time points. D1 and D2 = day 1 and 2 in the graph. The graph is representative of average ± SEM from three independent experiments and shows one way ANOVA. Significant difference between control and bacteria treatment is represented by *p<0.05 and significant difference between the scratch edge and other regions of a particular treatment group for the same time point is represented by +p<0.05. For a particular region significant between *P. gingivalis* and *F. nucleatum* is represented by ++p<0.05.**C.** Annexin V staining for assessing apoptosis in scratch assay cells. Cells were challenged with bacteria for 24 h and then stained with anti Annexin V antibody to visualize apoptosis. Cell membranes showing staining for Annexin V were considered positive for apoptosis. **D.** Graph representing Annexin V positive cells in the three regions (R1, R2 and R3) of the wound; values are average ± SEM from three independent experiments. Significant difference between control and bacteria treatment is represented by *p<0.05, +p<0.05 represents difference between scratch edge and other regions for a particular treatment group, ++p<0.05 denotes significant difference between *P. gingivalis* and *F. nucleatum* for a region.

Re-epithelialization is influenced by the rate of apoptosis, proliferation and migration [16], [17]. We examined each of these parameters. *In vitro* wounding of primary keratinocytes was performed and the impact of bacteria on keratinocyte apoptosis was examined by the TUNEL assay (Figure 2A) at a) the scratch edge (R1); b) in an area near the scratch edge (R2); and c) in an area at least 200 µm away from the scratch edge (R3). Creation of a scratch per se had a negligible effect on apoptosis which was similar at the scratch edge, near the scratch and away from it (see control, Figure 2A). *P*. *gingivalis* increased apoptosis by approximately ten fold at the scratch edge (Figure 2B), and two to five fold away from the scratch (p<0.05) (Figure 2A, 2B). *P*. *gingivalis* generally stimulated apoptosis to a greater extent than *F. nucleatum* (p<0.05). Typically the effect of both bacteria on apoptosis was greater at the scratch edge (Figure 2B) compared to being further away. Comparison of cell death due to bacteria at the scratch edge versus adjacent and far away regions showed significant difference with *P*. *gingivalis* (p<0.05) and *F. nucleatum* (p<0.05). Thus it appears that there is a spatial component when analyzing the effect of bacteria on cell death.

To further support the observation on cell death, scratch assay cells challenged with bacteria for 24 h were stained with anti-Annexin V antibody. *P*. *gingivalis* challenged cells showed higher number of Annexin positive staining in the cell membranes in all the three regions of the scratch compared to untreated control (p<0.05) (Figure 2C, 2D). Comparison between the different regions of the scratch showed that *P*. *gingivalis* showed significant difference between scratch edge and other regions (p<0.05). *F. nucleatum* had weaker effect on the scratched cells and showed significantly lower cell death compared to *P*. *gingivalis* in R1 and R3 (p<0.05). Thus the spatial effect of bacteria on cell death in wound healing model was confirmed by TUNEL and Annexin V staining.

### Bacteria Challenged Cells Exhibit Slow Rate of Cell Proliferation

**Figure 3:**
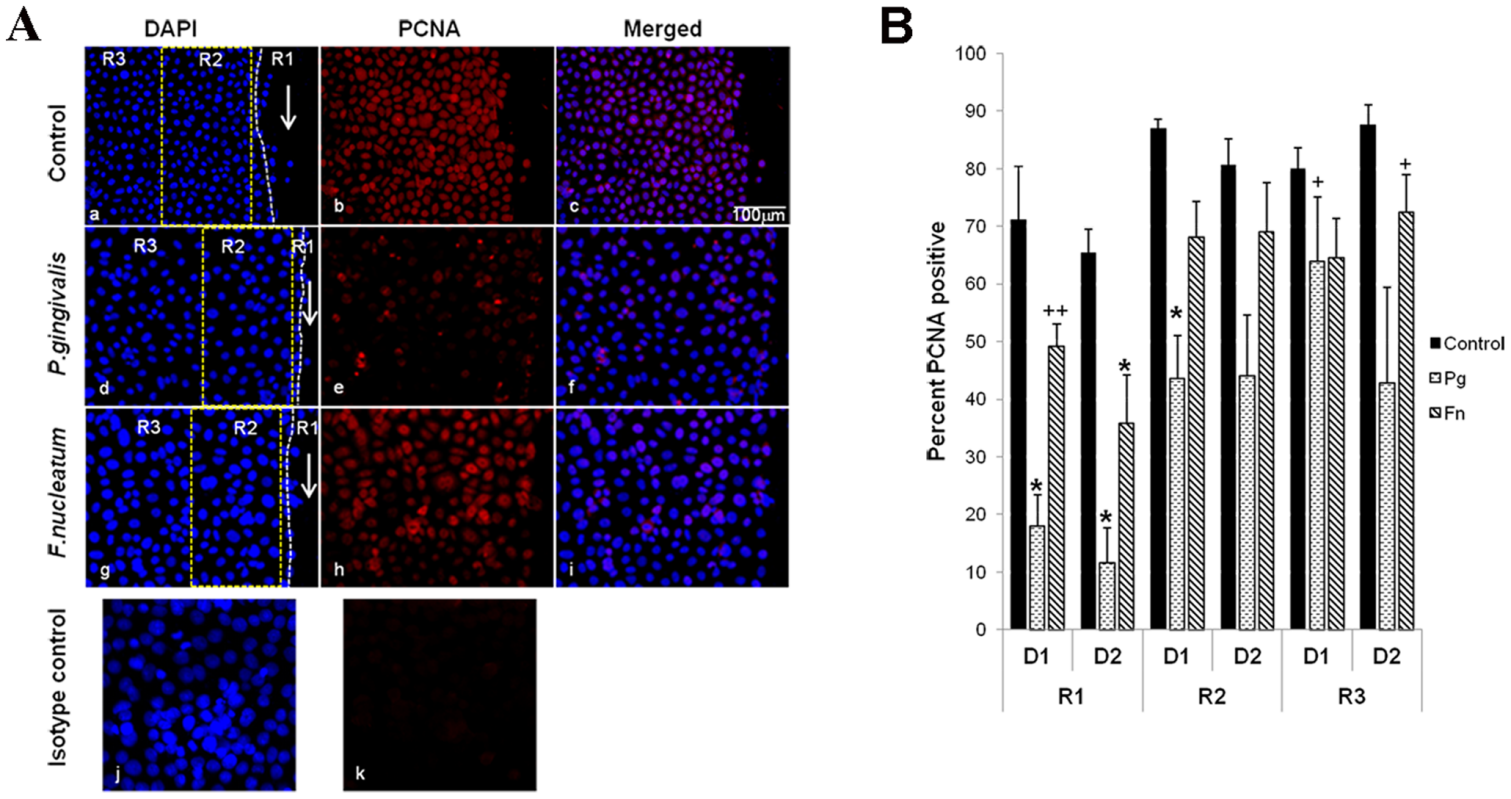
Assessment of cell proliferation in bacteria infected oral keratinocytes. **A.** Cell proliferation during wound healing was visualized using antibody against PCNA (b, e and h) and total number of cells in the field were marked by staining nuclei with DAPI (a, d and g). In primary gingival epithelial keratinocytes cell proliferation was assessed based on PCNA staining in regions R1, R2 and R3. The isotype control on bacteria challenged scratch assay for PCNA staining is represented by (k) with (j) showing DAPI staining. **B.** Graph representing cell proliferation at different regions of the scratch and at different time points. The total number of cells was counted based on DAPI staining of nuclei and cell proliferation was counted based on positive PCNA staining. D1 and D2 = day 1 and 2 in the graph. Average was calculated from three independent experiments ± SEM and shows one way ANOVA. Significant difference between control and *P. gingivalis* or *F. nucleatum* is represented by *p<0.05, scratch edge and other regions of a treatment group for the same time point is represented by +p<0.05. For a particular region significant between *P. gingivalis* and *F. nucleatum* is represented by ++p<0.05.

The effect of bacteria on cell proliferation was assessed in similar fashion as cell death, and regions R1, R2 and R3 were used for spatial analysis. Briefly, three distinct regions of the wounded area with increasing distance from site of injury was assessed for cell proliferation based on positive PCNA staining of nucleus. Incubation with *P*. *gingivalis* or *F. nucleatum* had a prominent impact on keratinocyte proliferation (Figure 3A). Keratinocytes at the scratch edge that were exposed to *P*. *gingivalis* had a 75%–80% reduction in the number of proliferating PCNA positive cells compared to cells without bacterial challenge (p<0.05) (Figure. 3B). As with apoptosis there also appeared to be a spatial effect as the impact of *P*. *gingivalis* on proliferation was greater at the scratch edge (p<0.05) (Figure. 3B). In the two areas further from the scratch edge *P. gingivalis* reduced the number of PCNA positive cells by 50% (p<0.05) (Figure. 3B). *F. nucleatum* challenged cells had 30% fewer PCNA positive cells at the scratch edge and 20% percent fewer PCNA positive cells in the two areas away from the scratch edge compared to cells not challenged with bacteria (p>0.05) (Figure. 3B). The greater impact of *P. gingivalis* compared to *F. nucleatum* was significant for scratch edge (p<0.05).

**Figure 4:**
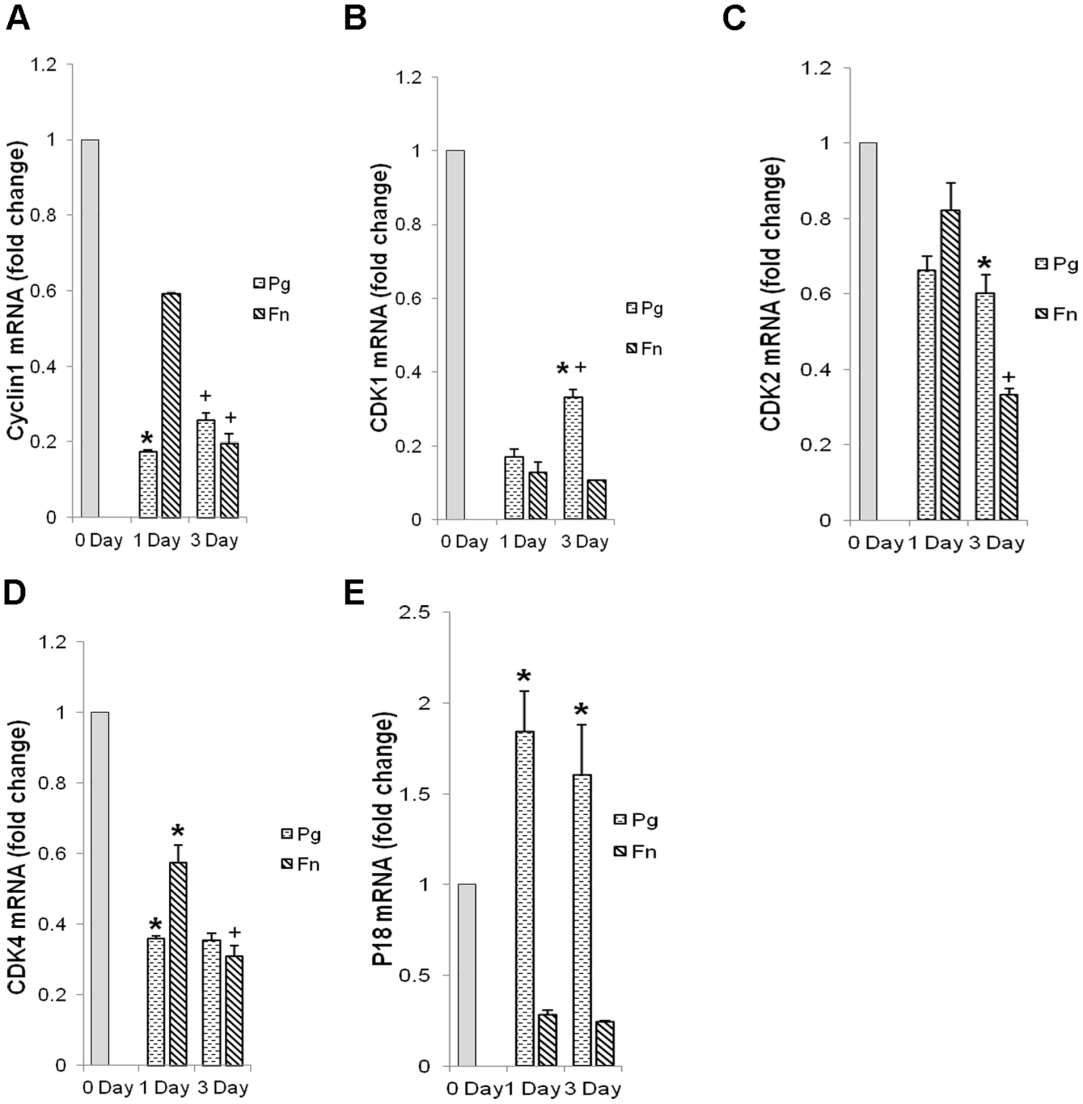
Analysis of expression of cell cycle genes in oral pathogen infected keratinocytes. Real time RT PCR of gene expression of Cyclin1 (A), cell division kinases 1, 2 and 4 (B, C, D) and cyclin dependent kinase inhibitor P18 (E) in bacteria challenged cells. Graph represents level of gene expression on day 1 and 3 post infection compared to day zero, i.e. prior to bacteria challenge. The data is average from three independent experiments. The δδCt gene/L32 value of each gene was calculated with normalization against expression of L32 control gene. Statistical significance represented by *p<0.05 and significant difference between expression on day 1 and 3 within a treatment group is represented by +p<0.05 after Tukey HSD test.

To further investigate the effect of bacteria on the proliferative behavior of keratinocytes mRNA levels of cell cycle genes were examined. *P. gingivalis* significantly downregulated the expression of cell cycle genes cyclin1, CDK1, CDK2 and CDK4 (p<0.05) by 30%–80% (Figure 4A, 4B, 4C, 4D). Similarly, *F. nucleatum* reduced cyclin1, CDK2 and CDK4 mRNA levels by 20%–90% percent (Figure 4A, 4C, 4D). In contrast the cyclin dependent kinase inhibitor P18 was found to be significantly upregulated by *P. gingivalis* (p<0.05) at all the time points while *F. nucleatum* reduced the same gene (Figure 4E).

### Cell Migration is Compromised in Bacteria Challenged Keratinocytes

**Figure 5:**
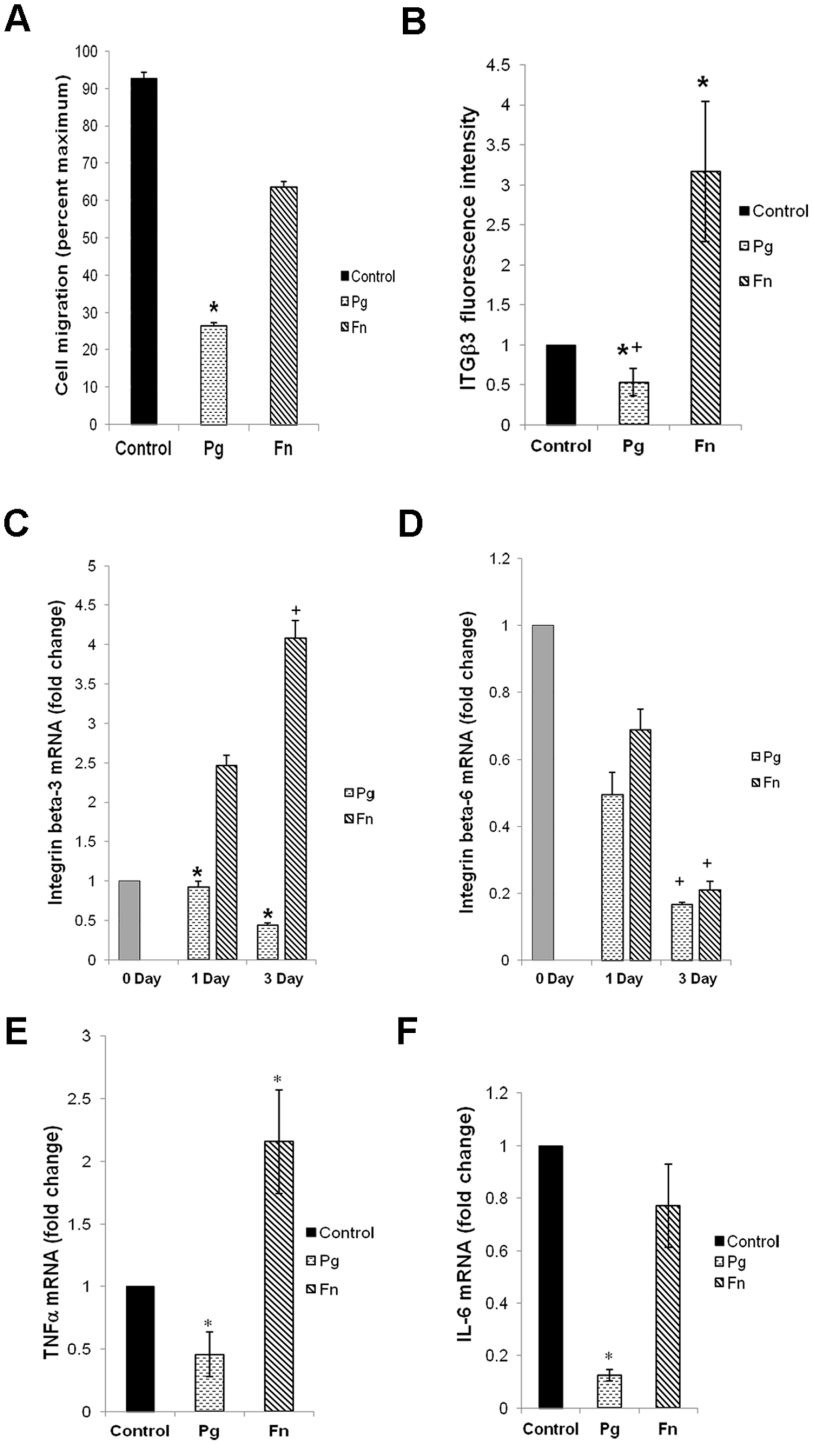
Study of cell migration and cytokine expression in pathogen infected oral keratinocytes. **A.** Cell migration was assessed using transwell migration of keratinocytes after incubation with bacteria. The number of cells was counted by DAPI staining of nuclei using NIS element software under fluorescence microscope. Values were expressed as percent maximum against untreated control. Data are means ± SEM and shows one way ANOVA, with level of significance *p<0.05. **B.** Quantitation of integrin beta-3 at the protein level was determined by measuring fluorescence intensity of integrin beta-3 normalized to cell numbers. Values represent means ± SEM from three independent experiments. Significant between control and treatment *p<0.05 and between the two treatment groups +p<0.05 was determined by Ttest. **C–D.** Gene expression analysis of integrin beta-3 and -6 on day 1 and 3 post infection by real time RT PCR. The data is average from three independent experiments, δδCt gene/L32 value of each gene was calculated with normalization against expression of L32 control gene. Statistical significance represented by *p<0.05, and significant difference between expression on day 1 and 3 in a treatment group is represented by +p<0.05 after Tukey HSD test. **E–F**. mRNA levels of TNFα and IL-6 were measured by real time RT-PCR. Data shown are mean from three independent experiments. Statistical significance in differences of expression against control is represented by *p<0.05 after Ttest.

To investigate the effect of bacteria on keratinocyte migration transwell assays were carried out. Incubation of oral keratinocytes with *P*. *gingivalis* reduced the number of cells that migrated by 73% compared to cells not challenged with bacteria (p<0.05), while *F. nucleatum* diminished cell migration by 36% (Figure 5A).

Since integrin beta-3 plays critical role in cell migration, further confirmation of the effect of oral bacteria on cell migration during wound healing was done by immunofluorescence quantitation. Fluorescence based protein expression study of integrin beta-3 in keratinocytes incubated with bacteria showed that *P*. *gingivalis* significantly reduced expression of pro migration protein by almost half (p<0.05) compared to control cells (Figure. 5B). *F. nucleatum* increased integrin beta-3 expression and differed from *P*. *gingivalis* by about six fold (p<0.05).

Bacteria modulated expression of migration associated genes such as integrin beta-3 and beta-6. Expression of integrin beta-3 was reduced by 56% (p<0.05) (Figure 5C) and integrin beta-6 was reduced by 83% (Figure 5D) upon incubation with *P*. *gingivalis* compared to cells not incubated with bacteria. The expression of integrins in *P*. *gingivalis* infected cells remained low even with progression of time. In contrast, *F. nucleatum* challenged cells had 300% increase in integrin beta-3 but had 78% decrease in beta-6 expression (Figure 5C, 5D). Thus, of the mRNA examined, *P*. *gingivalis* had a more consistent effect of downregulating mRNA levels of genes that promote migration of keratinocytes compared to *F. nucleatum*.

### Infection by Bacteria Alters Expression of Cytokines in Oral Keratinocytes

Differences in stimulation of TNFα were also noted; *P*. *gingivalis* reduced expression of TNFα by 50% (p<0.05) and *F. nucleatum* increasing it two fold compared to untreated control (p<0.05) (Figure 5D). Expression of IL-6 was downregulated by *P*. *gingivalis* by more than fivefold (p<0.05) while the same was marginally reduced by *F. nucleatum,* thus indicating that the two oral pathogens had varied effect on cytokine expression in infected cells.

## Discussion

Epithelial cells represent an important barrier and contribute to the host defense. The oral epithelium is continuously subjected to wear and tear due to daily activities. Breakage of the epithelial barrier represents vulnerable site for pathogen invasion. Oral bacteria can potentially affect the healing process following disruption of the mucosal barrier. We examined *P. gingivalis* and *F. nucleatum* because there have been several previous reports examining their impact on keratinocytes, particularly their inflammatory response and because they are frequently found in patients who have periodontal surgery [5], [9], [18]. Both *P. gingivalis* and to a lesser extent *F. nucleatum* caused a significant impediment to re-epithelialization when confluent cultures of keratinocytes were disrupted. The effect of bacteria persisted even though bacteria were removed from the cell cultures after 24 h and then incubated with media containing antibiotics. Labeled intracellular oral bacteria was detected 9 days after an overnight exposure suggesting that its long-lasting effect was due, in part to bacterial internalization. The internalization of *P. gingivalis* has been proposed to represent one of its pathogenic mechanisms [19], [20]. Proteolytic enzymes produced by bacteria represent a virulence factor [21], [22]. To test the role of gingipains in re-epithelialization, cells were co-incubated with bacteria and leupeptin, an inhibitor of serine and cysteine proteases. Leupeptin accelerated coverage of the denuded area by keratinocytes incubated with P. *gingivalis* but did not completely restore to the levels of cells that were not exposed to bacteria; a lag of 11% was observed. As a control we also demonstrated that incubation with leupeptin did not affect internalization of *P. gingivalis* or *F. nucleatum*.

The rate of re-epithelialization is affected by keratinocyte apoptosis, proliferation and migration. *P. gingivalis* and *F. nucleatum* had a significant impact on apoptosis. Interestingly, the effect was greatest at the “wound” edge suggesting that there is a synergy between mechanical cellular trauma and the impact of bacteria. Incubation with bacteria also had a negative impact on keratinocyte proliferation, which also had a spatial distribution with the effect being greatest at the “wound” edge. Our results contrasts with a report that *P. gingivalis* infected gingival keratinocytes have enhanced proliferation compared to uninfected keratinocytes [23]. The difference between our study from those reported by Kuboniwa and colleagues may be due to the fact that ours was examined under wound healing conditions.

To assess the impact on migration a transwell assay was used which occurs over a brief, 6 h period. *P. gingivalis* very substantially reduced the capacity of keratinocytes to migrate, which is a critical feature in the wound healing response [24]. *F. nucleatum* had a reduced effect that was still significant. Analysis of integrin beta-3 expression both at the transcript and protein level showed that *P. gingivalis* causes inhibitory effect on cell migration.

During wound healing cells divide, proliferate and migrate to cover the wound area and restore normal cell layers. Genes that are essential for re-epithelization of wounded region were affected by *P. gingivalis*. Progression through the mammalian cell cycle is governed by cyclins, cyclin-dependent kinases, and regulators such as cyclin dependent kinase inhibitors. In mammalian cells transient rise in levels of cyclin proteins leading to activation of Cdks are critical as Cyclin-CDK complexes trigger the transition to mitotic phase [25], [26]. Gene expression analysis showed downregulation of both cyclins and Cdks upon infection with oral pathogens *P. gingivalis* and *F. nucleatum*. Further, infection by *P. gingivalis* induced the expression of cell cycle-dependent kinase inhibitor P18 thereby indicating interference with expression of CDKs and inhibiting progression of cell cycle.

Further *P. gingivalis* increased the expression of cyclin dependent kinase inhibitor P18 suggesting block in progression to cell division. A possible explanation for the change in gene expression in keratinocytes caused by *P. gingivalis* infection is that successful persistence of pathogen is possible in non dividing host cells. Oral epithelium and epidermis normally lack beta integrin expression but wounding results in *de novo* synthesis and upregulation of integrins and involves expression of novel integrins such as beta-6 integrin that facilitates cell adhesion and migration [27], [28]. Upon wounding beta-3 integrin is reported to support actin cytoskeletal reorganization, polarization of cells and provides directionality to migration during wound healing [29]. There is paucity of information about the effect of oral pathogen on expression of integrins and in this study we observed that *P. gingivalis* downregulated the induction of integrin beta-3 and -6 in oral keratinocytes indicating impairment in cell-cell interactions and migration thereby obstructing healing. In comparison *F. nucleatum* had mild effect on healing. Overall *P. gingivalis* induced upregulation of cyclin dependent kinase inhibitor P18 on one hand while on the other it downregulated the induction of migration associated genes. Thus the aggressive influence of *P. gingivalis* on all the aspects related to oral wound healing can be attributed to the fact that the pathogen acts at the molecular level on genes that are critical for cell division and migration. Comparison amongst the effect of pathogens on expression of proinflammatory genes showed that *P. gingivalis* significantly reduced mRNA levels of TNFα and while *F. nucleatum* increased it.

Studies directed at understanding the effect of anaerobic periodontal pathogens suggest that bacteria invade mucosal cells, reside within cellular compartments, and impair restoration of normal oral tissue by interfering with cellular migration and proliferation [30], [31]. All of these aspects interfere with wound healing. In summary, from the current study it is clear that *P. gingivalis* and to a lesser extent *F. nucleatum*, two important oral bacteria impaired re-epithelialization in an *in vitro* scratch wound. This occurred through mechanisms that involved enhanced apoptosis, reduced migration and decreased proliferation. The capacity of these bacteria to specifically affect cellular processes vital for wound healing sheds light on how bacteria can affect the oral cavity. This study showed the long term effect of the pathogen after brief exposure. The model presented in the study mimics situations of *P. gingivalis* infection on oral wound due to mechanical injury such as would occur during trauma or resulting from periodontal or oral surgery. This is especially relevant for patients with periodontitis. Furthermore the effect was relatively long-term after a short incubation period with bacteria. Thus, early treatment of oral wounds may be particularly important in patients with diminished wound healing responses and in those who are susceptible to bacterial infection. Periodontal pathogens are associated with biofilm formation [32] and this aspect has not been addressed in our model. Besides this, the model of wound healing in this study is limited, as factors such as role of salivary defense proteins in *in vivo* have not been included. Thus, additional in vivo studies would be necessary to determine the impact of oral bacteria on healing wounds *in vivo*.
